# Heterologous prime-boost vaccination based on Polymorphic protein D protects against intravaginal *Chlamydia trachomatis* infection in mice

**DOI:** 10.1038/s41598-022-10633-x

**Published:** 2022-04-22

**Authors:** Romina Cecilia Russi, Diego Del Balzo, Agustín Luján, Ivana Gabriela Reidel, María Inés García, Carolina Veaute, María Teresa Damiani

**Affiliations:** 1 Biochemistry and Immunity Laboratory, School of Medicine, University of Cuyo, IMBECU-CONICET, Centro Universitario, 5500 Mendoza, Argentina; 2 Experimental Immunology Laboratory, School of Biochemistry and Biological Sciences, National University of Litoral, Ciudad Universitaria, 3000 Santa Fe, Argentina

**Keywords:** Vaccines, Vaccines

## Abstract

The control of the worldwide spread of sexually transmitted *Chlamydia trachomatis* (*Ct*) infection urgently demands the development of a preventive vaccine. In this work, we designed a vaccine based on a fragment of polymorphic protein D (FPmpD) that proved to be immunogenic enough to generate a robust systemic and mucosal IgG humoral immune response in two strains of mice. We used a heterologous prime-boost strategy, including simultaneous systemic and mucosal administration routes. The high titers of anti-PmpD antibodies elicited by this immunization scheme did not affect murine fertility. We tested the vaccine in a mouse model of *Ct* intravaginal infection. Anti-PmpD antibodies displayed potent neutralizing activity in vitro and protective effects in uterine tissues in vivo. Notably, the humoral immune response of PmpD-vaccinated mice was faster and stronger than the primary immune response of non-vaccinated mice when exposed to *Ct*. FPmpD-based vaccine effectively reduced *Ct* shedding into cervicovaginal fluids, bacterial burden at the genitourinary tract, and overall infectivity. Hence, the FPmpD-based vaccine might constitute an efficient tool to protect against *Ct* intravaginal infection and decrease the infection spreading.

## Introduction

More than a million sexually transmitted infections (STI) are acquired every day worldwide. *Chlamydia trachomatis (Ct),* the most frequent sexually transmitted bacteria, are responsible for substantial morbidity and a high economic burden on public health. In 2012, the WHO estimated 131 million chlamydial infections of the 357 million STI cases^1^. *Ct* infection displays an asymptomatic silent course in 30–50% of men and 70–90% of women, and consequently, it remains undiagnosed and untreated^2^. Chronic chlamydial infection may cause severe complications in the woman’s upper reproductive tract, including pelvic inflammatory disease (PID), ectopic pregnancy, endometritis, and infertility^3^. In the male genitourinary tract, *Ct* infections generate urethritis and inflammation of the accessory glands, which may impair male fertility^4^. Consequently, it is necessary to develop a vaccine to prevent the spreading of *Ct* infections^5^. Despite the many efforts made throughout more than seventy years, unfortunately, no preventive vaccines against *Ct* are available^6^.

*Ct* vaccine design with a rational selection of antigen, formulation, and administration scheme is a great challenge. Partially, the pathogenesis of *Ct* at the genital tract is related to the consequences of the immune response induced by the infection^7^. Therefore, the goal is to develop a vaccine efficient for generating a protective immune response with no associated immunopathogenesis. Numerous attempts, using diverse vaccine strategies, assaying different immunogens, antigen types, routes of vaccine administration, and adjuvants have been done^6^. In preclinical and clinical trials, heterologous prime-boost schemes have proven to constitute successful approaches for protection against different infectious diseases^8^. Despite the advantages of this vaccination strategy, it has not been widely and comprehensively investigated for *Ct* yet.

Among the antigen candidates evaluated for a chlamydial vaccine, members of the Polymorphic membrane protein family (Pmps) have arisen as promising components. Pmps are autotransporter-like immunogenic surface-exposed proteins. They play a role as adhesins and display potent antigenic properties^9–11^. PmpD is an attractive vaccine candidate for the prevention of *Ct* infections due to its interstrain conserved nature, surface localization, implications in early host-cell interaction, and immunological importance because it is the target of neutralizing antibodies^9,12,13^. As previously shown, antibodies essentially contribute to the resolution of primary *Ct* infections, and prevent bacterial systemic dissemination in mice^14,15^. Furthermore, studies from antibody-deficient mice revealed the fundamental role played by the humoral immune response in conferring protection against chlamydial genital tract re-infection^16,17^. Lately, immunity against *Ct* infection mediated by protecting antibodies has attracted renewed attention^18^.

A crucial feature for chlamydial infections is that vaccination should induce systemic and mucosal immunity at the genital tract. Particular characteristics of the genital tract are the lack of mucosal-associated lymphoid tissues, the influence of the hormonal cycle, and that IgG is the dominant immunoglobulin, in contrast to other mucosal tissues where the dominant class is IgA^19–21^. Immunization at one mucosal immune inductive site may generate immunity locally and at a distant mucosa^22^. Intranasal immunization stimulates nasal-associated lymphoid tissues and evokes IgG and IgA antibody response in both the respiratory and the female genital tract^23,24^. More importantly, intranasal immunization generates memory T cells residing in the genital mucosa, which might contribute to the prevention of chlamydial re-infection^25,26^.

In this work, we selected a fragment of PmpD (FPmpD: aa693-aa1240) that displays three regions with B- and T-cell epitopes identified by in silico prediction^13^. Furthermore, this study is the first to evaluate this FPmpD fragment as a vaccine candidate for preventing *Ct* infections. We hypothesized that anti-FPmpD antibodies might mediate vaccine-induced protection against intravaginal *Ct* challenge. Therefore, we designed a PmpD-based vaccine using the heterologous prime-boost strategy, including systemic and mucosal administration. Then, in a murine model of genital *Ct* infection, we analyzed the protective efficacy and adverse effects of the vaccine. Our findings indicate that the present formulation and scheme of immunization induce a protective immune response against intravaginal *Ct* infection.

## Results

### *Ct* PmpD fragment induced systemic and cervicovaginal anti-PmpD antibodies in two mouse strains

**Figure 1:**
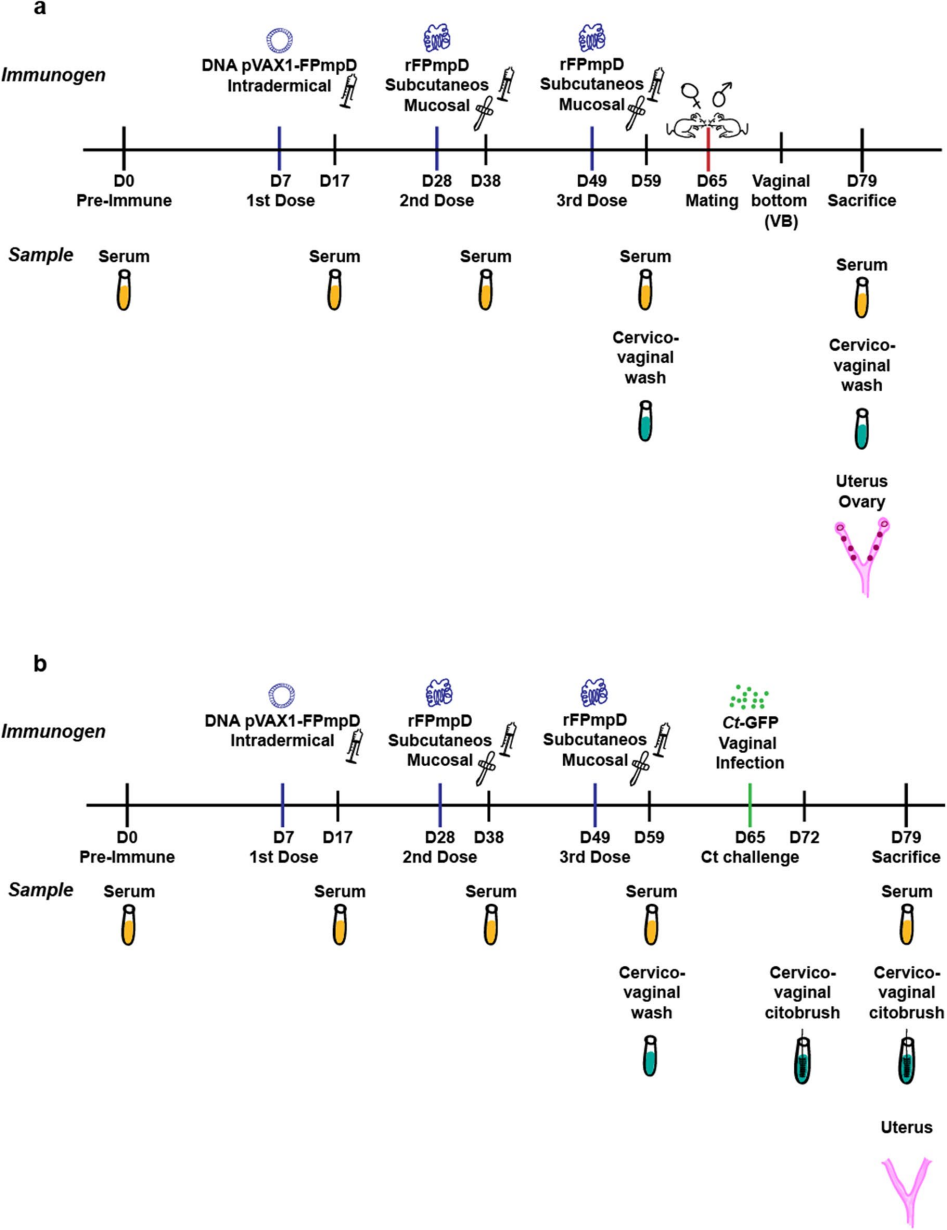
A timeline representing the experimental protocol for BALB/c (**a**) and C57BL/6 (**b**) mice. A schematic representation of the immunogen and administration route used for immunization, and the type of sample obtained at each time point. D indicates the experimental day.

**Figure 2:**
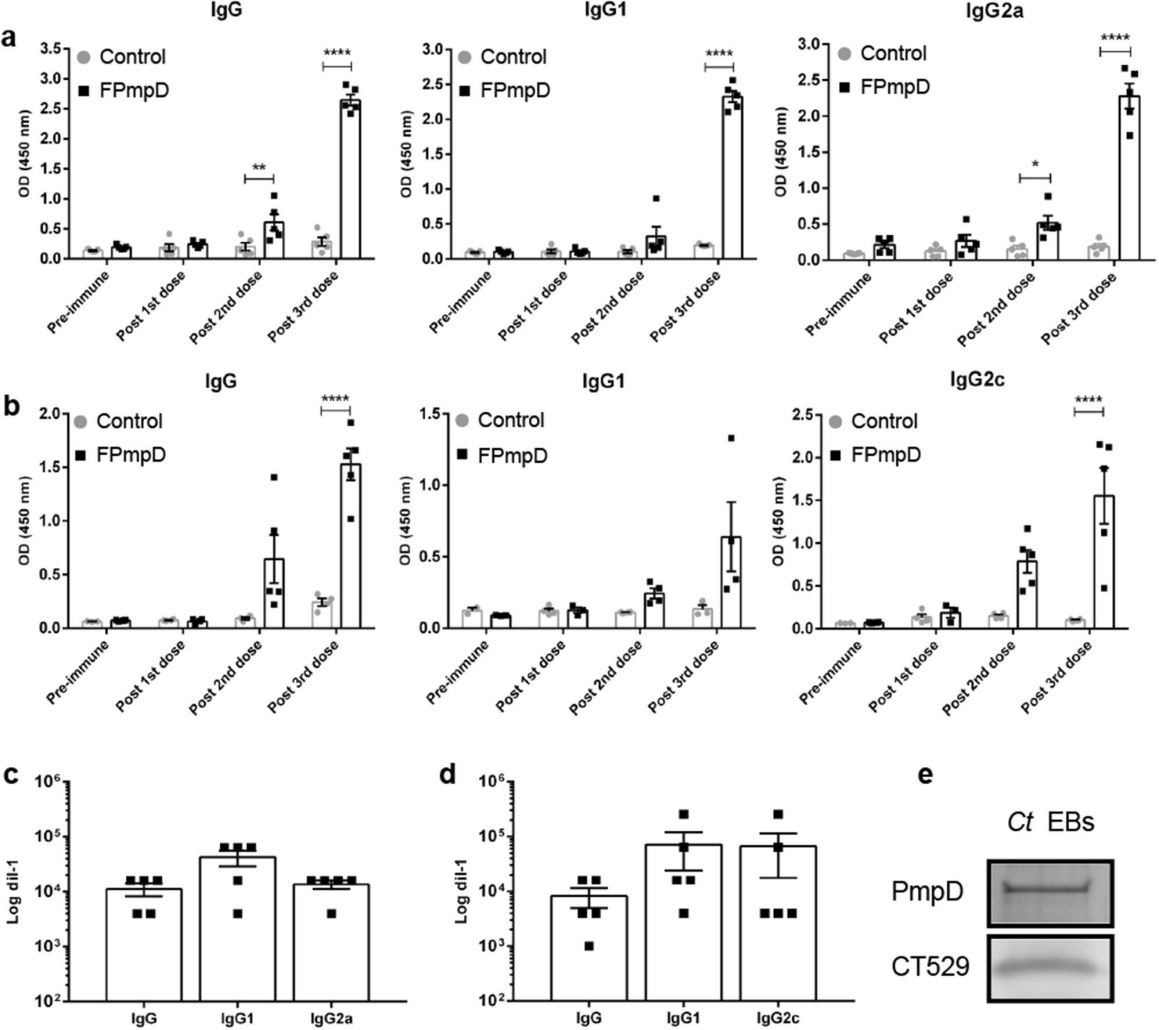
Systemic humoral immune response. (**a**,**b**) Anti-PmpD IgG, IgG1, IgG2a/c antibodies in sera from non-vaccinated (control) and FPmpD-vaccinated (FPmpD) BALB/c (**a**) and C57BL/6 (**b**) mice, ten days after each dose. Each dot represents the mean OD value of each mouse serum evaluated by duplicate. Bars represent means ± SEM for each experimental group (1:100 dilutions; two-way ANOVA with Sidak post-test; **p < 0.01; ****p < 0.0001). (**c**,**d**) Specific anti-PmpD IgG, IgG1 and IgG2a/c titration of BALB/c (**c**) and C57BL/6 (**d**) sera obtained ten days post last dose, respectively. Bars indicate means ± SEM. (Anova with Tukey’s post-test). (**e**) Reactivity against *Ct* of a pool of sera (1:50) obtained ten days post last dose from FPmpD-vaccinated mice (PmpD) by immunoblotting. Anti-CT529 rabbit policlonal IgG was used as a control for the detection of *Ct* in the lysate (CT529).

**Figure 3:**
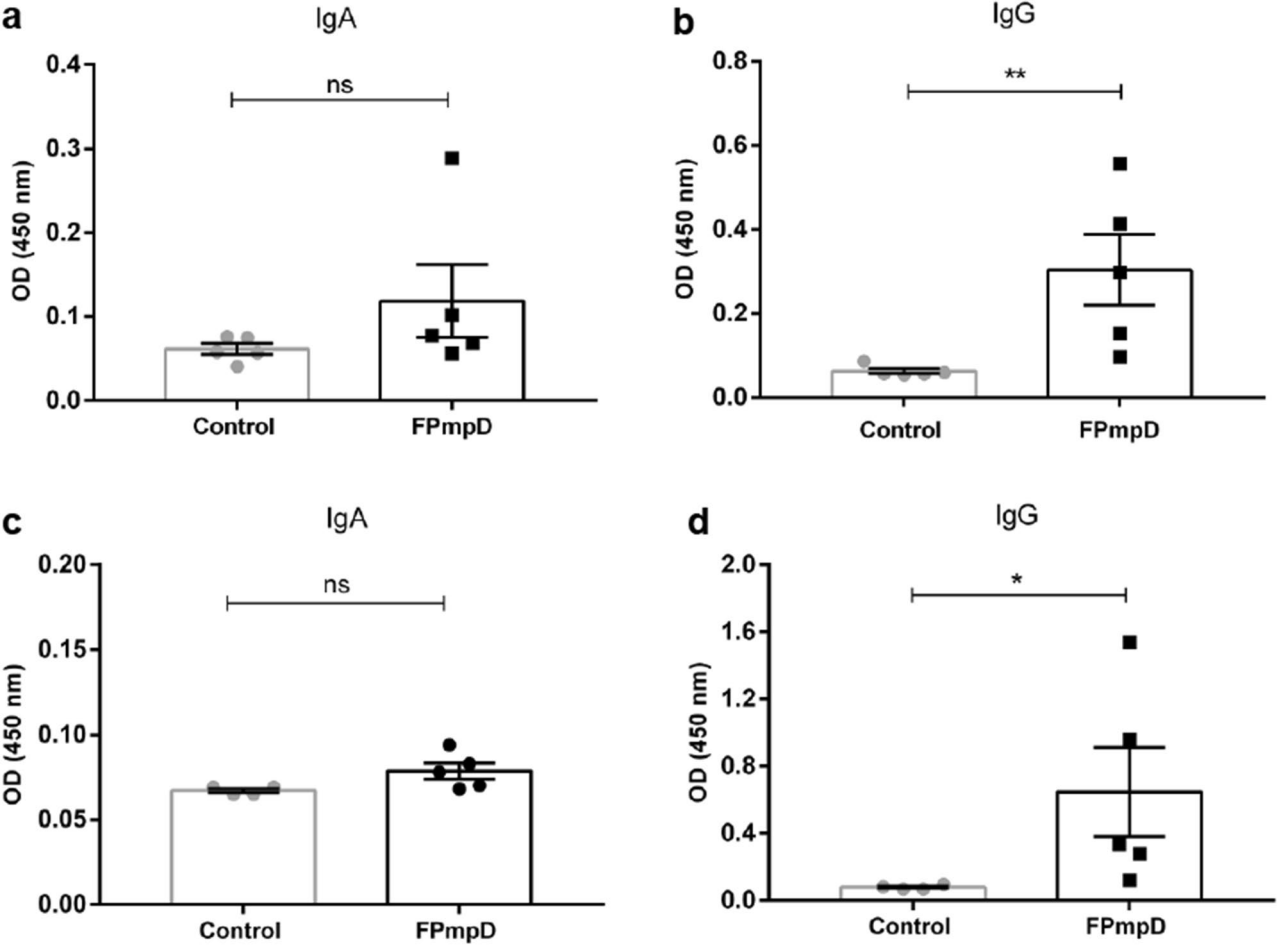
Vaginal humoral immune response. Anti-PmpD IgA and IgG antibodies in vaginal washes obtained ten days post last dose from non-vaccinated (Control) and FPmpD-vaccinated (FPmpD) BALB/c (**a**,**b**) and C57BL/6 (**c**,**d**) mice. Bars indicate means ± SEM for each experimental group (1:3 dilutions; two-way ANOVA with Sidak post-test; *ns* no significant differences; *p < 0.05; **p < 0.01).

We assessed the immunogenicity of the PmpD fragment (FPmpD) in two strains of mice immunized using a prime-boost strategy with DNA (FPmpD-pVAX1) followed by two doses of the recombinant protein (rFPmpD) as indicated in “Methods” (Fig. 1). For the evaluation of the systemic humoral immune response, we measured anti-PmpD IgG, IgG1, and IgG2a/c levels in serum before immunization (pre-immune), or ten days after each dose in BALB/c (Fig. 2a,c) and C57BL/6 (Fig. 2b,d) mice. The prime-boost vaccine strategy with FPmpD induced demonstrable increments of specific IgG, IgG1, and IgG2a/c antibodies with successive immunizations that became significant after the second dose in BALB/c mice (p < 0.01). Noticeably, the third dose led to a further significant increase in antibody levels in both mice strains (p < 0.0001) (Fig. 2a,b). There were no specific anti-PmpD IgG, IgG1, and IgG2a/c in pre-immune sera and ten days after the first dose in both groups. The titer of anti-PmpD IgG, IgG1, and IgG2a/c antibodies in serum samples ten days post last dose (Fig. 2c,d) were between 10^4^ and 10^5^ dils^−1^ for animals of both strains. To corroborate the reactivity of the anti-PmpD antibodies to non-conformational epitopes on elementary bodies of purified bacteria (*Ct* EBs), we performed an immunoblot using pooled sera obtained ten days post last dose (Fig. 2e). The sera from the vaccinated mice strongly reacted against *Ct* EBs. The presence of chlamydial proteins in the lysate was confirmed by detecting the inclusion protein CT529 with rabbit polyclonal antibodies (Fig. 2e and Supplementary Fig. 1). Next, we investigated the ability of the FPmpD vaccine to generate mucosal humoral immunity at the genital tract. We measured anti-PmpD IgA and IgG antibodies in cervicovaginal washes obtained ten days after the last immunization. Although the FPmpD vaccine triggers anti-PmpD IgA synthesis at the genital tissues of some mice, the mean IgA humoral immune response did not significantly differ from non-immunized control BALB/c and C57BL/6 mice, respectively (Fig. 3a,c). In contrast, we observed a robust IgG immune response in cervicovaginal washes from vaccinated BALB/c (Fig. 3b) and C57BL/6 (Fig. 3d) mice. Our findings indicate that FPmpD triggers a potent systemic and mucosal IgG humoral immune response.

### FPmpD prime-boost immunization generated neutralizing antibodies that reduce Ct infectivity in vitro

**Figure 4:**
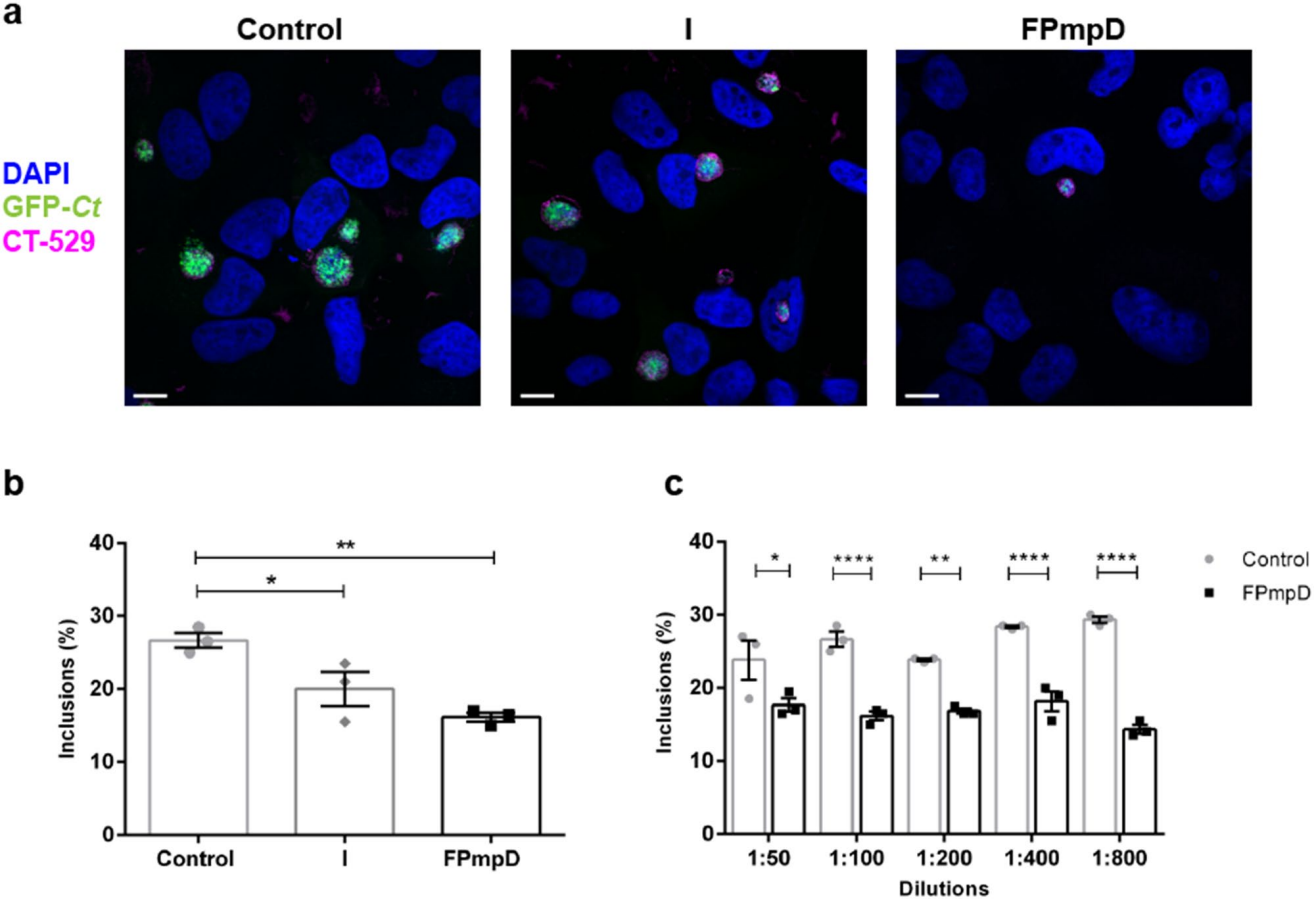
In vitro neutralization assay. (**a**) Confocal images of HeLa cells infected with GFP-*Ct* previously incubated with sera from non-vaccinated uninfected (Control), non-vaccinated infected (I), and FPmpD-vaccinated uninfected (FPmpD) mice. Nuclei were labeled with DAPI, chlamydial inclusion membrane were detected with anti-CT-529 antibodies, and bacteria expressed the green fluorescent protein (GFP-*Ct*). Bar represents 10 µm. (**b**) Quantification of the number of GFP-*Ct* inclusions by confocal microscopy. Each dot represents the mean of each group evaluated by triplicates. At least 540 cells were evaluated for each replication and experimental condition. Results are expressed as number of inclusions per 100 cells (%). Bars indicate means ± SEM for each experimental group (one-way ANOVA; Dunnet post-test; *p < 0.05; **p < 0.01). (**c**) Number of GFP-*Ct* inclusions developed in the presence of different dilutions of sera from non-vaccinated uninfected (Control) and FPmpD-vaccinated uninfected (FPmpD) mice. Each dot represents the mean of each group evaluated by triplicate. Six different fields were counted for each replication and condition. Results are expressed as number of inclusions per 100 cells (%). Bars indicate means ± SEM for each experimental group (1:50, 1:100, 1:200, 1:400, 1:800 dilutions; Mann–Whitney; *p < 0.05; **p < 0.01; ****p < 0.0001).

We investigated the neutralization ability of anti-PmpD antibodies from sera of vaccinated mice. Briefly, GFP-*Ct* were incubated with mice sera and then used to infect HeLa cells. Inclusions were visualized by GFP fluorescence and by staining the inclusion membrane with anti-CT529 antibodies. DAPI labeled bacterial and eukaryotic DNA. We analyzed sera from 3 groups of mice: non-vaccinated uninfected (Control), non-vaccinated infected (I), and FPmpD-vaccinated uninfected (FPmpD) mice. Figure 4a shows representative confocal images of every experimental group. Next, we quantified the number of inclusions, counting at least 540 cells per condition. We observed a marked decrease in the number of inclusions developed per 100 cells in the presence of sera from FPmpD-vaccinated mice versus non-vaccinated ones. Besides, the neutralizing ability of antibodies elicited by vaccination was higher than antibodies produced after chlamydial infection in non-vaccinated mice (Fig. 4b). Moreover, serial dilutions of sera showed a 30 to 50% reduction in the number of inclusions per 100 cell developed by in vitro infection of HeLa cells when sera belonged to FPmpD-vaccinated (FPmpD) mice compared to non-vaccinated (Control) ones (Fig. 4c). Altogether, these results indicate that the FPmpD prime-boost vaccine generates neutralizing antibodies that decrease chlamydial infectivity in vitro.

### FPmpD prime-boost immunization did not alter mice fertility

**Figure 5:**
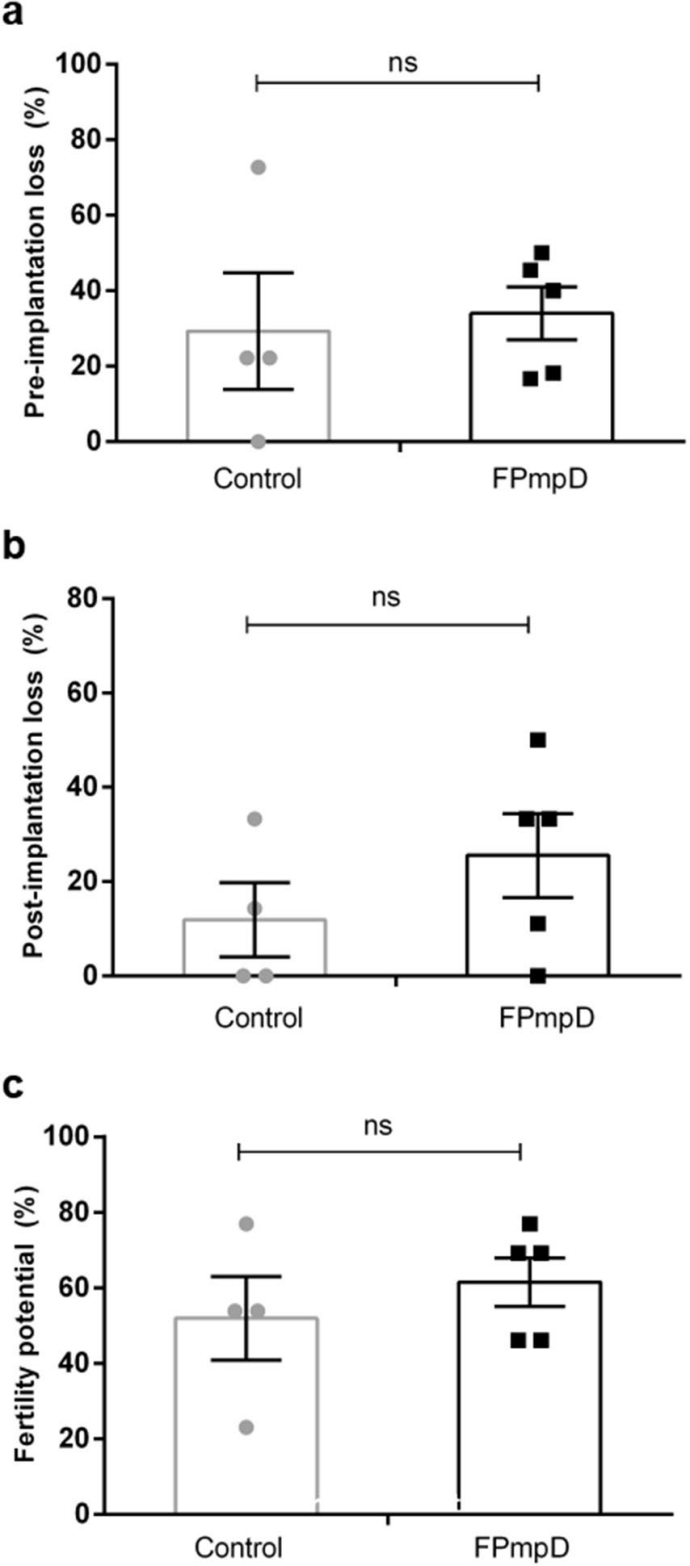
Fertility assay. In FPmpD-vaccinated (FPmpD) and non-vaccinated (Control) mice we evaluated: (**a**) Pre-implantation loss [(number of corpora lutea − number of implantations)/number of corpora lutea] × 100. (**b**) Post-implantation loss [(number of implantations − number of live fetuses)/number of implantations] × 100. (**c**) Fertility potential [implantation sites/corpora lutea] × 100. Each dot represents individual mouse values. Bars represent means ± SEM value for each experimental group (two-way ANOVA; Sidak post-test; *ns* non-significant differences).

Next, we evaluated the impact of FPmpD prime-boost immunization on the fertility of mice since a robust immune response in mucosa may affect it^27^. We determined the number of corpora lutea, implantion sites, resorptions, live and dead fetuses in vaccinated and non-vaccinated control mice. The fertility potential, assessed as indicated in “Methods”, showed the implantation efficiency and threw similar results after FPmpD vaccination (FPmpD) compared to non-vaccinated (Control) mice (Fig. 5a). Besides, we calculated the rate of pre-implantation loss from the number of corpora lutea and implantation sites; and the rate of post-implantation loss by assessing the number of implantation sites and live fetuses, as indicated in “Methods”. No significant differences in the pre-implantation (Fig. 5b) and post-implantation (Fig. 5c) loss rates were observed between vaccinated and non-vaccinated mice, indicating that FPmpD prime-boost strategy does not impair fertility.

### FPmpD prime-boost immunization reduced bacterial burden at the genital tract and infectivity after the *Ct* challenge

**Figure 6:**
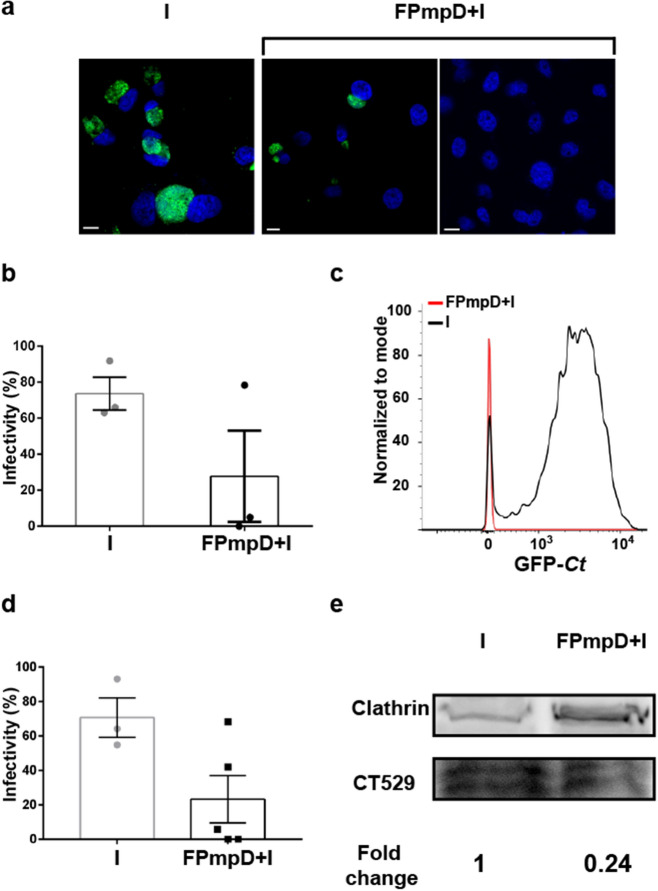
Infectivity assay. HeLa cells were incubated with cervicovaginal secretions collected seven days post GFP-*Ct* challenge from non-vaccinated (I) and FPmpD-vaccinated (FPmpD + I) mice. At 24 h post infection, cell cultures were fixed and inclusions developed by infective bacteria shed in vaginal fluids were detected by confocal microscopy (**a**,**b**) and by flow cytometry (**c**,**d**). (**a**) Confocal images show the number and size of chlamydial inclusions developed by incubation with cervicovaginal secretions from one non-vaccinated infected mouse (I, left panel) and two FPmpD-vaccinated infected mice (FPmpD + I, middle and right panels). (**b**) Infectivity was calculated as the percentage of HeLa cells infected with cervicovaginal secretions from non-vaccinated infected (I) and from FPmpD-vaccinated infected (FPmpD + I) mice. A minimum of 200 cells were counted for each condition. (**c**) A representative flow cytometry profile indicating the normalized green fluorescence intensity that corresponds to GFP-*Ct* developed in HeLa cells incubated with cervicovaginal secretions from a non-vaccinated infected mouse (I, black line) or from a FPmpD-vaccinated infected mouse (FPmpD + I, red line). (**d**) Percentage of HeLa cells infected with cervicovaginal secretions from non-vaccinated infected (I) and from FPmpD-vaccinated infected (FPmpD + I) mice. At least 10,000 HeLa cells were analyzed per condition. Each dot represents the percentage of cells infected by the cervicovaginal secretion of each mouse. Bar represents means ± SEM of infected cells of each experimental group. (**e**) Detection of *Ct* proteins in uterine homogenates from non-vaccinated infected (I) and FPmpD-vaccinated infected (FPmpD + I) mice analyzed 14 days post-infection by immunoblot with anti-CT529 antibodies. Clathrin was used as loading control.

We analyzed the impact of the FPmpD-based vaccine on *Ct* shedding into cervicovaginal fluids and the infectivity of these samples. Thus, we collected cervicovaginal samples from non-vaccinated (I) and FPmpD-vaccinated (FPmpD + I) mice seven days post-GFP-*Ct* challenge. *Ct* burden in cervicovaginal fluids correlates with overall sample infectivity assessed on an in vitro cell culture-based assay. We analyzed infectivity by confocal microscopy (Fig. 6a,b) and flow cytometry (Fig. 6c,d) and expressed it as the percentage of HeLa cells infected by the sample. Figure 6a shows representative confocal images from HeLa cells infected with cervicovaginal samples collected from non-vaccinated infected (I) (left panel) and FPmpD-vaccinated infected (FPmpD + I) (middle and right panels) mice. The FPmpD-vaccine strongly reduced the bacterial burden in cervicovaginal fluids. In agreement, it decreased, or in most cases, suppressed samples infectivity. Despite in the FPmpD-vaccinated group, we observed a null percentage of infected cells in almost all mice; we detected the presence of a limited infection in one mouse. In contrast, the samples from the non-vaccinated group of mice challenged with *Ct* generated numerous and large chlamydial inclusions when assayed on HeLa cells. A quantification of confocal images is shown in Fig. 6b. A representative profile displaying the green fluorescence associated with GFP-*Ct*, assessed by flow cytometry, is shown in Fig. 6c. It shows the level of infection of HeLa cells after incubation with cervicovaginal samples from a vaccinated mouse and a non-vaccinated one; after challenging both mice with *Ct*. We found no infected HeLa cells (0–2%) in three of five FPmpD-vaccinated mice. One mouse from the vaccinated group displayed lower infectivity than the mean of non-vaccinated mice. And, the sample from only one vaccinated mouse showed similar infectivity than those from the non-vaccinated group. Figure 6d shows the percentage of HeLa cells infected with the cervicovaginal secretions from non-vaccinated (I) and FPmpD-vaccinated (FPmpD + I) mice assessed by flow cytometry. Although differences are no statistically significant, the results clearly show that vaccination decreased *Ct* shedding into cervicovaginal fluids and mice infectivity.

In line with these findings, we speculated a reduced chlamydial burden in the uterine tissue from FPmpD-vaccinated mice intravaginally infected with *Ct*. At 14 days post-GFP-*Ct* challenge (sacrifice day), we couldn`t find *Ct* in the cervicovaginal samples of any mice, neither by confocal microscopy nor by flow cytometry (data not shown). Therefore, we decided to analyze the presence of *Ct* in the uteri of non-vaccinated (I) and FPmpD-vaccinated (FPmpD + I) infected mice. We detected the chlamydial protein CT529 by immunoblotting in pools of uterine samples of both groups as a marker of bacterial replication in genital tissues. In agreement with our previous findings, higher levels of CT529 were detected in uteri from non-vaccinated (I) compared to FPmpD-vaccinated (FPmpD + I) mice (Fig. 6e and Supplementary Fig. 2). These findings suggest that FPmpD-vaccinated mice have more efficient chlamydial clearance, reducing bacterial shedding into cervicovaginal fluids; and decreased *Ct* burden in the uterus, likely preventing the advance of the infection towards the upper genital tract.

### FPmpD prime-boost immunization protected against chlamydial infection and resulted in decreased pathology at the genital tract

**Figure 7:**
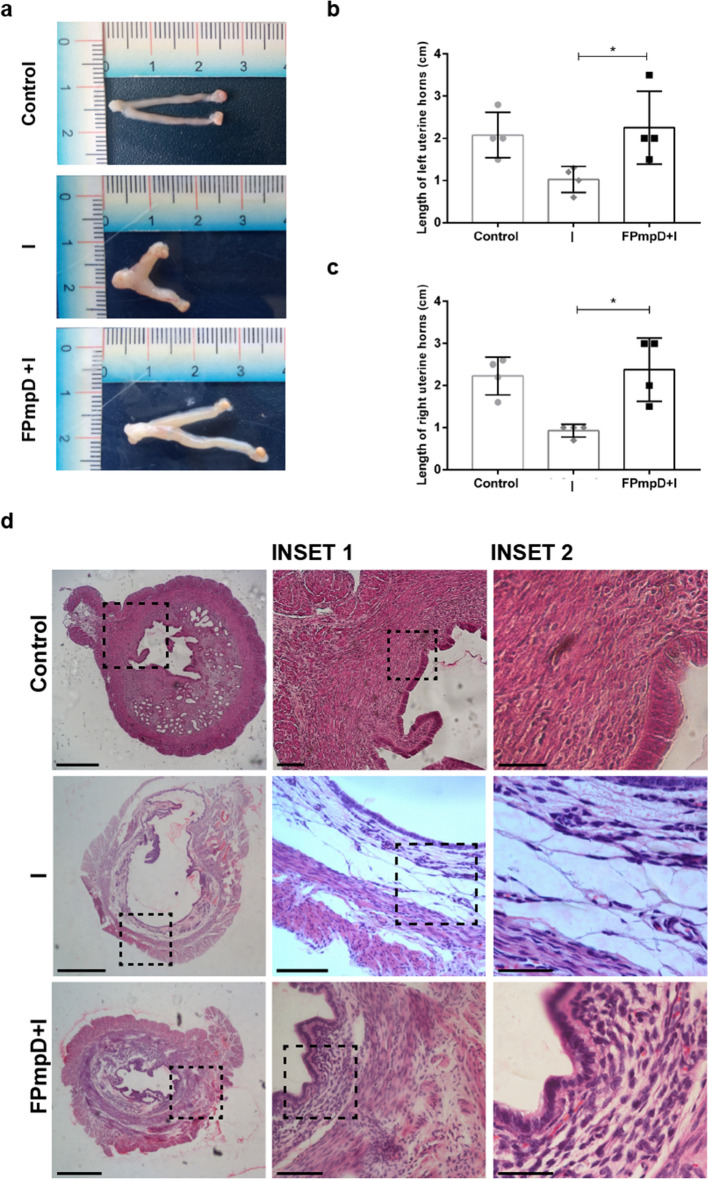
Morphological and Histological analysis of uterine horns. (**a**) Representative images of uteri from non-vaccinated infected mouse (I), FPmpD-vaccinated infected mouse (FPmpD + I), and non-vaccinated uninfected (Control) mice. (**b**,**c**) Length of left (**b**) and right (**c**) uterine horns. Each dot represents an individual mouse. Bars represent means ± SEM of each experimental group (Mann–Whitney; *p < 0.05). (**d**) Representative hematoxylin–eosin stained horn sections from non-vaccinated infected (I), FPmpD-vaccinated infected (FPmpD + I), and non-vaccinated uninfected (Control) mice on day 14 post *Ct* infection. Middle and right panels are magnifications of the insets of the selected areas of the corresponding images.

*Ct* infection provokes inflammation, edema, hyperemia, and diverse pathological lesions as hydrosalpinx or tubal stenosis at the genital tract, which ultimately can lead to irreversible damage of reproductive tissues and infertility^28^. Therefore, we next evaluated the efficacy of the FPmpD-based vaccine to prevent histopathological damage at the genital tract of vaccinated mice after *Ct* infection. As indicated in “Methods”, the genital tract from each mouse was obtained 14 days after the *Ct* challenge to evaluate morphological and histological aspects. Representative images of uteri from the different experimental groups are shown in Fig. 7a. A morphological feature caused by *Ct* infection is the shortening of the uterine horns of infected mice^29^. Accordingly, *Ct* infection significantly shortened uterine horns from non-vaccinated (I) mice compared to FPmpD-vaccinated (FPmpD + I) ones that displayed horns of similar length to uninfected non-vaccinated (Control) mice (Fig. 7b,c). Images show that vaccination not only prevented horns shortening but also reduced morphological alterations caused by *Ct* infection. At the histological level, representative images show that *Ct* infection altered oviduct lumen size and distorted the tissue architecture. Non-vaccinated infected (I) mice displayed dilated oviduct lumens compared to FPmpD-vaccinated infected (FPmpD + I) mice, which showed a similar oviduct lumen size to uninfected non-vaccinated (Control) mice. Neutrophil infiltration was not evident in *Ct*-infected uterine tissues. Besides, these representative images show edema and loss of tissue structure in uteri from non-vaccinated infected (I) mice while vaccination (PmpD + I) prevented the tissue damage induced by the infection. Moreover, vaccination itself did not produce histological alterations of the uterine tissue, although the high titers of specific antibodies elicited (Fig. 7d). Altogether, our findings indicate that the FPmpD-based vaccine efficiently reduced the chlamydial burden at uterine tissues, and congruently, it decreased morphological and histopathological alterations of the genital tract associated with *Ct* infection.

### FPmpD prime-boost immunization generated anti-PmpD antibodies sustained over time and elicited a strong humoral immune response after the *Ct* challenge

**Figure 8:**
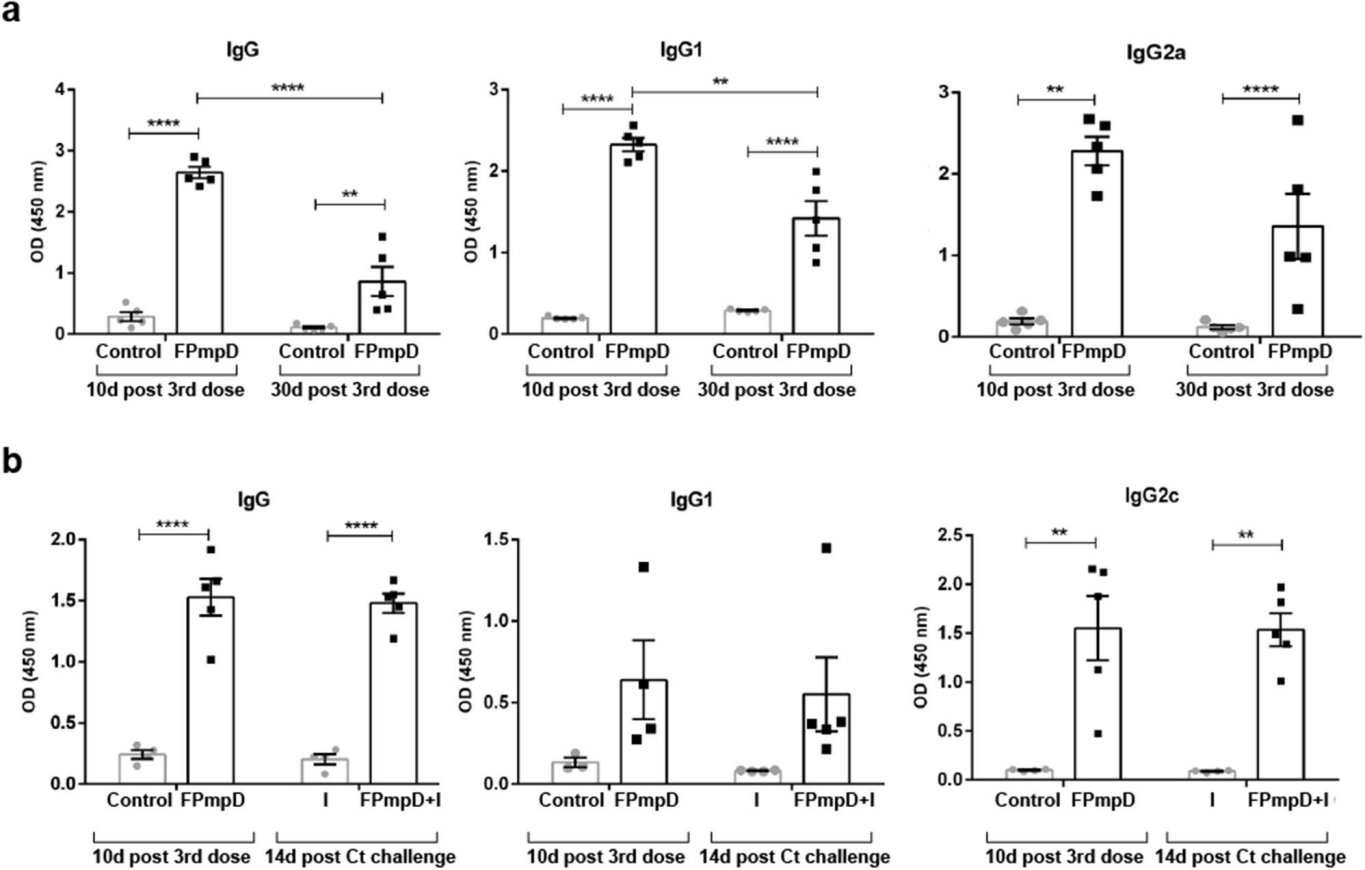
Evaluation of the humoral immune response over time. (**a**) Anti-PmpD IgG, IgG1, IgG2a antibody levels in sera obtained at the indicated times from non-vaccinated (Control) or FPmpD-vaccinated (FPmpD) BALB/c mice. (**b**) Anti-PmpD IgG, IgG1, IgG2c antibody levels of sera obtained ten days post last dose from non-vaccinated (Control) and FPmpD-vaccinated (FPmpD) C57BL/6 mice; and in sera collected 14 days post the *Ct* challenge from non-vaccinated infected (I) and FPmpD-vaccinated infected (FPmpD + I) C57BL/6 mice. Each dot represents the mean OD value of each individual mouse evaluated by duplicate. Bars represent the means ± SEM for each experimental group (1:100 dilutions; two-way ANOVA with Sidak post-test; **p < 0.01, ****p < 0.0001).

A fundamental condition that a vaccine should accomplish is to generate memory lymphocytes able to drive a consistent and fast immune response when challenged by the pathogen. In this sense, we analyzed the ability of the FPmpD-based vaccine to trigger the production of specific antibodies along the time; and the humoral immune response elicited after the *Ct* challenge. By ELISA, as indicated in “Methods”, we determined anti-PmpD antibodies at ten and 30 days after the 3rd dose, the last one of the FPmpD-based vaccine. Although there was a slight decrease in antibody levels between day ten and day 30 post 3rd dose, at both time points, we found that FPmpD-vaccinated mice (FPmpD) exhibited significant high levels of specific antibodies. Furthermore, IgG1 and IgG2a antibody levels at 30 days post 3rd dose remained elevated compared to non-vaccinated uninfected mice (Control) (p < 0.001) (Fig. 8a). These findings indicated a sustained humoral immune response after vaccination, even in the absence of *Ct* challenge.

Next, we evaluated the production of anti-PmpD antibodies in the group of challenged animals. In vaccinated infected (FPmpD + I) mice, the levels of antibodies 14 days after *Ct* intravaginal infection were similar to those obtained ten days after the 3^rd^ dose in the same animals (Fig. 8b). Conversely, the primary infection itself did not elicit significant anti-PmpD antibodies, exhibiting antibody levels similar to non-vaccinated uninfected mice. Thus, vaccination produced a fast and robust humoral immune response after the *Ct* challenge, substantially higher than the one triggered by the primary infection itself (Fig. 8b). Altogether, our results show that FPmpD-vaccinated mice produced systemic antibodies that remained elevated over time and elicited a strong humoral immune response when mice were infected, likely consistent with the generation of memory T cells.

## Discussion

Improvements in screening programs and the availability of a preventive vaccine constitute the actual challenge in fighting against the spreading of chlamydial infections in undeveloped and developed countries. All over the world, scientists have conducted extensive efforts to generate an effective *Ct* vaccine, which protects against both infection and genital tract pathology^27,28^. Different chlamydial antigens have been assayed as vaccine candidates. Among them, members of the polymorphic membrane proteins (Pmps) family. Pmps participate in the adhesion and entry to host cells and have potent immunogenic properties, triggering the immune response in humans and mice^30^. Notably, a vaccine composed of the recombinant PmpD N-terminal protein fragment generated a strong humoral immune response, which conferred protection to mice against chlamydial vaginal infection^31^. In the current study, we designed a vaccine based on another fragment of PmpD involving three regions bearing B- and T-cell epitopes by in silico prediction^13^. Given the PmpD conserved sequence among serovars, we proposed a formulation based on this antigen to develop a broadly protective vaccine covering different circulating *Ct*^18^. Owing to PmpD participation in bacterial entry to the host cells, we hypothesized that PmpD-based vaccine might generate neutralizing antibodies able to block chlamydial attachment to cells. At the same time, this vaccine formulation would prevent infection from spreading. In agreement with our hypothesis, our results showed that FPmpD-based vaccine triggered high titers of systemic IgG, IgG1, and IgG2a/c antibodies without the addition of any adjuvant. A robust humoral immune response was elicited after the third dose, and it remained consistently elevated the following 30 days in two strains of mice, BALB/c and C57BL6. Importantly, we found that anti-PmpD antibodies possess strong neutralizing properties, substantially decreasing the chlamydial infection in vitro in a cell culture-based assay*.*

The antibody-mediated protection has been extensively characterized in mouse models of *Chlamydia muridarum* infection, and lately, increasing data have highlighted a role for antibodies in vaccine-mediated protection against *Ct* infections, the resolution of the primary infection, and prevention of reinfection^17,31–33^. A vaccine scheme based on recombinant major outer membrane protein (MOMP) without adjuvant elicited a robust humoral immune response that efficiently protects against chlamydial genital infections^34^.

In the present work, we used a heterologous prime-boost regimen for immunization with PmpD antigen. Reports have shown that using plasmid DNA for priming allows the activation of cell-mediated immunity, required for memory B cells generation. Then, the protein boost consolidates the specific immune response and induces the differentiation of such B cells in antibody-secreting cells, leading to a robust humoral immune response^8,35^. Successful results have been reported using heterologous prime-boost vaccines against *Plasmodium falciparum* and *Mycobacterium bovis*^36–38^. In the case of *Ct*, a vaccine based on MOMP DNA priming followed by a MOMP protein booster was efficient to clear intravaginal infection^39^.

The route of vaccine administration is an important issue to generate immune protection at the infection gate. *Ct* vaccine should produce an efficient immune response at the genital mucosa. It has been well established that intranasal immunization is an effective route for antigen delivery to induce a strong humoral immune response and resident memory T cells at the genital mucosa^25,40^. To promote both systemic and mucosal humoral responses, we chose a vaccination scheme in which mice were immunized by intradermal, subcutaneous, and intranasal routes. Our study demonstrated the efficacy of the FPmpD prime-boost vaccine with simultaneous immunization routes to elicit a robust systemic and mucosal antigen-specific IgG immune response. To simplify the immunization scheme while vaccine efficacy is not affected, future studies should determine the contribution to the immunity of each immunization route separately, as well as analyze a homologous prime-boost regimen.

It is important to highlight that, both at the mouse and human female genital tract, the dominant immunoglobulin isotype is IgG rather than secretory IgA^20,21^. Furthermore, it has been shown that specific Th1-associated IgG2c antibodies generated by the rectal administration of a chlamydial vaccine prevail over IgA in cervicovaginal secretions, and they exert a protecting effect against genital infection in mice^41^. Besides, IgG-mediated protection is an effector mechanism relevant in other sexually transmitted genital infections^42,43^. The FPmpD-based vaccine elicits specific IgG2a/c isotype antibodies, which have been previously associated with the induction of a protective Th1 profile, leading to bacterial clearance at genital tissues and the overall resolution of infection^44^. Consistent with our findings, other studies that evaluated combined immunization strategies obtained improvements in mucosal response, high levels of IgG and IgA at the genital tract, and protection against an intravaginal challenge with *Ct*^14,18,45^.

In the current study, we focused on developing a vaccine with the ability to substantially induce neutralizing antibodies against PmpD that protect against *Ct* intravaginal infections without causing adverse effects at the genital tract. We demonstrated that FPmpD-vaccinated mice had similar fertility parameters to non-vaccinated mice, notwithstanding the high titers of anti-PmpD antibodies elicited at the cervicovaginal mucosa. *Ct* have been detected in the cervix, endometrium, and fallopian tube of women with endometritis, salpingitis, and pelvic inflammatory disease. A common pathological feature is the fallopian tube inflammation caused by chlamydial infections, which ultimately can lead to chronic pelvic pain, tubal obstruction, ectopic pregnancy, and infertility^46,47^. Therefore, the *Ct* vaccine should not trigger inflammatory mechanisms that can damage reproductive tissues^27^. Further, antibodies have been linked to protection against upper genital pathology^48,49^. In our work, uteri from non-vaccinated mice displayed typical macro and microscopical signs of chlamydial infection like horns shortening, enlargement of the lumen, and edema. In contrast, the FPmpD-based vaccine prevented the alteration of the architecture of tissues from the murine genital tract when challenged with *Ct.* In agreement, our findings suggest that the in vitro neutralization effect of anti-PmpD antibodies correlates with in vivo protection at the genital tract of FPmpD-vaccinated mice. Actually, the FPmpD-based vaccine reduced the chlamydial burden at uterine tissues, decreased chlamydial shedding in cervicovaginal fluids, and in definitive, efficiently reduced the infectivity of murine vaginal secretions.

Another interesting issue is that the intravaginal *Ct* challenge of vaccinated mice elicited a faster and more robust humoral immune response with the production of higher levels of anti-PmpD antibodies compared to the primary infection of non-vaccinated animals, suggesting the induction of a memory immune response.

Altogether our findings provide fundamental insights for anti-chlamydial FPmpD-based vaccine development. Further studies should be conducted to simplify the immunization strategy and to evaluate the effectiveness of the FPmpD-based vaccine in humans. Taken together, current data envision this vaccine as a promissory candidate to advance in the fight against chlamydial infections.

## Methods

### Cell culture and bacterial strains

Bacteria used were *Chlamydia trachomatis* serovar L2 434/Bu (*Ct*) (gently given and typified by Unidad de Estudios de Clamidias, UBA, Bs. As., Argentina); serovar D (generously provided by Instituto Malbran, Bs. As., Argentina); and, a green fluorescent *Ct* L2 strain harboring p2TK2-SW2 IncDProm-RSGFP-IncDTerm serovar L2 (GFP-*Ct*) kindly provided by Derré^50^. Bacteria were propagated in HeLa 229 or McCoy cells, harvested, purified, and quantified as previously described by Del Balzo et al.^51^. HeLa cells (ABAC, Argentina) were cultured in high glucose Dulbecco´s Modified Eagle´s Medium (GIBCO, Thermo Fisher Scientific, Argentina) supplemented with 10% (v/v) fetal bovine serum (FBS) (Internegocios SA, Argentina), 0.3 mg/mL l-glutamine (ICN Biomedicals Inc) and 1.55 mg/mL glucose (Biopack, Argentina) without antibiotics in 5% CO2 at 37 °C.

### Cloning of FPmpD and plasmid purification for DNA immunization (pVAX1-FPmpD)

The FPmpD insert was obtained by PCR from genomic DNA of McCoy cells infected with *Ct* serovar D, using specific oligonucleotides designed from the sequence AF268092.2 of the L2 serovar, using the Primer 3 Plus software^52^. The FPmpD coding sequence preceded by the human α1-antitrypsin signal peptide was inserted into the gene immunization plasmid (pVAX1). A synthetic gen (SG) was obtained by de novo synthesis with specific oligonucleotides designed using the DNA works software v3.2.4^53^. These oligonucleotides overlap each other and contain a 20 base pairs (bp) complementary region to pVAX1 downstream from the *Eco*RI cleavage site, the Kozak sequence [5'ACCATGG3 '], the coding sequence for the human α1-antitrypsin signal peptide, and the first complementary 19 bp coding for the PmpD fragment (Table 1). Both the FPmpD and the SG sequences were cloned into the pVAX1 vector using the SLiCE method^54^. Finally, the SLiCE product (pVAX1-FPmpD) was used to chemically transform *E. coli* DH5α cells^55^. The PCR and the assembly products, as well as the pVAX1-FPmpD plasmid, were evaluated by agarose gel electrophoresis, PCR, and DNA sequencing. To generate the plasmid for immunization, *E. coli* Top 10 cells transformed with pVAX1-FPmpD were cultured in 200 mL of Luria Bertani (LB) broth in the presence of Kanamycin, at 37 °C, 18 h. Subsequently, the plasmid was recovered by alkaline lysis and purified by organic extraction with phenol and chloroform. The same procedure was performed for the plasmid without insert (pVAX1).

**Table 1:** Primer sequences used for cloning.

**FPmpD sintetic gen**
pVAX1-k (Sense primer) **CCACTAGTCCAGTGTGGTGG***ACC*
pVAX1-k-SP (Antisense primer) TGCCAGCAGGAGGATGCCCCACGAGACAGAAGACGG*TGCCATGGT***CCACCACACTGGACT**
SP-FPmpD (Sense primer) TGCCAGCAGGAGGATGCCCCACGAGACAGAAGACGGTGCCATGGTCCACCACACTGGACT
SP-FPmpD (Antisense primer) CTCCACGTAAGCAAGAAATAGCCAGGGA
**FPmpD**
Sense primer ATTTCTTGCTTACGTGGAGATG
Antisense primer **TGCTGGATATCTGCAGAATT*CTA***CTGAGCAGTGAGATTATGAGCAA

Regions in bold hybridize to pVAX1, Underlined regions hybridize to the Ct-PmpD sequence. Italicized regions correspond to the kozak sequence (k). The unmodified regions correspond to the signal peptide (SP). Bold italic regions correspond to the stop codon.

### Cloning, expression, and purification of the recombinant PmpD protein fragment (rFPmpD)

A DNA sequence encoding FPmpD was inserted into the pET 32(a) vector (Merck Millipore, Germany) with a C-terminal hexahistidine tag. *E. coli* BL21 (DE3) cells transformed with the plasmid construction pET32a-FPmpD were grown 18 h in LB medium, supplemented with 0.1 mg/mL of ampicillin, at 37 °C, with shaking. Protein expression was induced overnight with 1 mM isopropyl-d-1-thiogalactopyranoside. The FPmpD recombinant protein (rFPmpD) was purified from the cell pellet with a Ni-nitrilotriacetic acid column (GE Healthcare Life Science, USA), as indicated by the manufacturer. rFPmpD concentration was measured by the Bradford’s method^56^ and the purity was analyzed by 15% sodium dodecyl sulfate polyacrylamide gel electrophoresis (SDS-PAGE) followed by Coomassie brilliant blue staining^57^.

### Mice

Six-eight weeks old female BALB/c mice belonging to Icivet Litoral, Argentina, and C57BL/6 female mice (The Jackson Laboratory, USA) were bred and maintained under specific pathogen-free (SPF) conditions (temperature 20–24 °C, humidity 50–60%, 60 air exchanges per hour in the cages, and a 12/12 h light/dark cycle with the lights on at 8 am). The maximum caging density was five mice from the same litter and sex starting from weaning. Mice were provided drinking water and a standardized mouse diet ad libitum. Animal handling was performed in accordance with the Guide for the Care and Use of Laboratory Animals (ILAR 2010). Animal protocols were approved by the Institutional Committee of Laboratory Animal Care and Use (CICUAL, UNCUYO, Argentina) and by the Institutional Advisory Committee on Research Ethics and Security (FBCB-UNL, Argentina). Mice were randomly divided into the different groups. As described in the Author contribution`s section, different researchers manipulate the animals, ones performed the immunizations while others infected the mice and acquired the data. This study is reported in accordance with ARRIVE guidelines (https://arriveguidelines.org).

### FPmpD-based immunization of female mice using a prime-boost strategy

Female BALB/c (n = 6) and C57BL/6 (n = 5) mice were immunized with a first ear-pinnae-intradermal dose of FPmpD-pVAX1 (50 μg in 20 μL of 0.05 M sterile sodium acetate buffer pH 5.3). Two rFPmpD boosters, were administered every three weeks, by intranasal (2.5 μg in 20 μL of sodium acetate buffer) and subcutaneous (5 μg in 100 μL of sodium acetate buffer) routes, simultaneously. The BALB/c (n = 5) or C57BL/6 (n = 4) control mice groups received the plasmid without insert (pVAX1) in the first dose and sodium acetate buffer without recombinant protein by the same immunization routes than the vaccinated mice in the second and third doses. Blood was collected from the submandibular vein ten days after each inoculation for anti-PmpD antibodies evaluation. Vaginal fluid (VF) was obtained by vaginal washing with PBS ten days after the last dose, and stored at − 80 °C with protease inhibitors cocktail Set I (Merck, Germany) until use. At the sacrifice, 30 days post last dose, mice were bled by cardiac puncture, under 100 mg/kg Ketamine (Hollyday-Scott SA, Argentina) and 10 mg/kg Xylacin (König SA, Argentina), and sera were store for anti-PmpD antibodies assessment. Timelines that represent experimental protocols are displayed in Fig. 1.

### Measurement of serum and cervicovaginal anti-PmpD antibodies

The humoral immune response to rFPmpD was evaluated by an indirect ELISA. Briefly, 96 well-polystyrene plates (Microlon™ 600, Greiner Bio-One, Austria) were coated with 1 μg/well of rFPmpD. After blocking non-specific binding sites with PBS supplemented with 5% skimmed milk, 1:100 dilutions of sera in PBS-1%-skimmed milk or 1:3 dilutions of VF were tested. Anti-FPmpD IgG, IgG1, IgG2a (BALB/c) or IgG2c (C57BL/6), and IgA were assessed by incubation with peroxidase-conjugated anti-mouse IgG (Jackson, USA), anti-mouse IgG1 (Santa Cruz Biotechnology, Inc., USA), anti-mouse IgG2a (Abcam Inc., USA), anti-mouse IgG2c (Abcam Inc., USA) or anti-mouse IgA (Sigma-Aldrich, USA) respectively, followed by incubation with hydrogen peroxide and tetramethylbenzidine (Zymed, USA). Optical Density (OD) at 450 nm was measured using a microplate reader (Mulstiskan EX, Labsystems, USA). The results are expressed as OD. The serum samples collected ten and 30 days post last dose from non-vaccinated (Control) and FPmpD-vaccinated (FPmpD) mice were titrated. The cut-off value was calculated as the average of the control group’s OD plus 3 standard deviations.

### Determination of neutralizing ability of anti-PmpD antibodies

Briefly, HeLa cells were grown to confluence in 96-well flat-bottom microtiter plates. The GFP-*Ct* (MOI: 2) were incubated for 30 min at 37 °C with different dilutions (1:50; 1:100; 1:200; 1:400; 1:800) of vaccinated (FPmpD) and non-vaccinated (Control) mice sera. Then, these bacteria were placed onto HeLa cells and incubated for 24 h. Inclusions were visualized with a confocal microscope Olympus FV-1000. Serum samples from vaccinated and non-vaccinated mice were assayed in triplicates. As positive controls of infection, equivalent amounts of GFP-*Ct* (incubated with sera from non-vaccinated uninfected mice) were used to infect HeLa cells in triplicates. Results were quantified as inclusion forming units (IFU). The assay was adapted from Olsen et al.^58^.

### Assessment of female mice fertility

Six-eight weeks old BALB/c female mice were mated with non-treated 14–16 weeks old male mice of proven fertility. In each box, three females were mated with one male after ten days of the last immunization dose. Animals were monitored daily for the presence of vaginal plugs or spermatozoa in the vaginal secretion; when detected, it was considered day 0 of pregnancy. The female mice were separated and kept until day 18 of pregnancy and then were sacrificed. After the collection of uteri and ovaries, we determined the number of corpora lutea, implants, resorptions, live and dead fetuses. According to Perobelli et al.^59^, we determined: 1. Fertility potential (efficiency of implantation): implantation sites/corpora lutea × 100; 2. Rate of preimplantation loss: [(number of corpora lutea − number of implantations)/number of corpora lutea] × 100; and 3. Rate of postimplantation loss: [(number of implantations − number of live fetuses)/number of implantations] × 100. The fertility was evaluated in non-vaccinated (control) and FPmpD-vaccinated (FPmpD) mice.

### Vaginal *Ct* challenge and determination of chlamydial shedding and infectivity

The mouse model of intravaginal infection was performed as extensively described by Lujan et al.^29^. *Ct* serovar L2 was used as indicated in Shaw et al.^60,61^. At day 16 after the last dose, FPmpD-vaccinated (n = 5) and non-vaccinated (n = 4) C57BL/6 mice were intra-vaginally challenged with 1.5 × 10^5^ GFP-*Ct* serovar L2 elementary bodies (EBs) as previously described^29^. To synchronize the murine estrous cycle, all C57BL/6 mice received a single subcutaneous injection of 2.5 mg of medroxyprogesterone acetate (Holliday-Scott SA, Argentina) 1 week before the GFP-*Ct* infection. Cervicovaginal swabs were obtained with disposable microapplicators (Multi-Brush, Denbur Inc, USA) at seven and 14 days post-challenge from FPmpD-vaccinated infected (FPmpD + I) and non-vaccinated infected (I) mice. Infectious bacterial progeny released in the cervicovaginal secretion was tested by confocal microscopy and by UFIs assay quantified by flow cytometry, as previously described by Vromman et al.^62^.

At the sacrifice, 14 days post-infection, mice were bled by cardiac puncture and serum aliquots were stored at − 20 °C until used for antibody analysis. To evaluate the presence of chlamydial proteins, uterine horn sections were stored using 100 µL of sterile PBS supplemented with the protease inhibitor cocktail p-2714 (Sigma-Aldrich, USA) at − 80 °C.

### Immunoblotting assays

After the *Ct* challenge, uterine horns from non-vaccinated infected (I) or FPmpD-vaccinated infected (FPmpD + I) mice were lysed in the presence of the protease inhibitor cocktail using tissue grinder and sonication to generate protein extracts for the determination of chlamydial proteins by immunoblotting. Equal amounts of proteins from each sample were resolved in 15% SDS-PAGE gels (n = 3). Then, proteins were transferred onto 0.45 µm nitrocellulose membranes (Amersham). After the Ponceau S staining, membranes were cut at 55 kDa and blocked in TBS/5% skimmed milk for one h at room temperature. Subsequently, the parts of the membranes with proteins of MW lower than 55 kDa were incubated overnight with rabbit anti-CT529 (1:100) antibodies generously given by Agathe Subtil (Pasteur Institute, Paris, France) followed by the corresponding goat anti-rabbit HRP-conjugated IgG (1:2500) secondary antibodies (Jackson ImmunoResearch Laboratories, Philadelphia, USA). The protein load was assessed in the part of the membranes with proteins of MW heavier than 55 kDa with rabbit anti-clathrin heavy chain antibody (1:1000) (Abcam, Cambridge, UK). Bound antibodies were revealed using the reagent ECL PlusTM (GE Healthcare Life Sciences) in an ImageQuant LAS4000. The intensity of the bands was quantified with the ImageJ software (Fiji) and expressed as arbitrary units. The reactivity against CT529 confirmed the presence of *Ct* in uterine tissues.

Lysates of *Ct* EBs separated by electrophoresis were transferred to nitrocellulose membranes (n = 3). After Ponceau S staining, membranes were cut at 55 kDa and blocked in TBS/5% skimmed milk for one h at room temperature. Then, the parts of the membranes with proteins heavier than 55 kDa were incubated with a pool of sera (1:50) from FPmpD-vaccinated (FPmpD) mice followed by anti-mouse HRP-conjugated IgG (1:2500) (Jackson ImmunoResearch Laboratories, Philadelphia, USA) and revealed by ECL. The parts of the membranes with proteins of MW lower than 55 kDa were incubated overnight with rabbit anti-CT529 (1:100) polyclonal antibodies (Pasteur Institute, Paris, France) followed by goat anti-rabbit HRP-conjugated IgG (1:2500) (Jackson ImmunoResearch Laboratories, Philadelphia, USA) and revealed by ECL (GE Healthcare Life Sciences).

### Histological and morphological analysis of uterine horns

At the sacrifice, 14 days post-infection, C57BL/6 mice were anesthetized and euthanized by cervical dislocation. The uterine horns were dissected for morphological and histological analysis. Uterine horns lengthof vaccinated (FPmpD + I) and non-vaccinated (I) infected mice was measured. One uterine horn of each mouse was fixed in 4% paraformaldehyde and embedded in paraffin according to standard procedures. Uterine horn slices (5 μm–thick) were stained with hematoxylin–eosin and examined with a Nikon 80I microscope equipped with a DS-QiMc camera for histological analysis. Images were processed using Nis elements F 3.2 software (Nikon, Japan).

### Statistical analysis

Results report the mean ± standard deviation of mean (SDM). Statistical differences among groups were analyzed by a two-tailed t-test or one-way and two-way ANOVA using GraphPad Prism 6.0 software (GraphPad, USA). P values less than 0.05 were considered statistically significant.

## Supplementary Information

Supplementary Information 1.

Supplementary Information 2.
