# Supplementary figures and images for: Heterologous prime-boost vaccination based on Polymorphic protein D protects against intravaginal Chlamydia trachomatis infection in mice
Source: Sci Rep. 2022 Apr 22;12:6664. doi: 10.1038/s41598-022-10633-x (PMC9030682; doi:10.1038/s41598-022-10633-x)

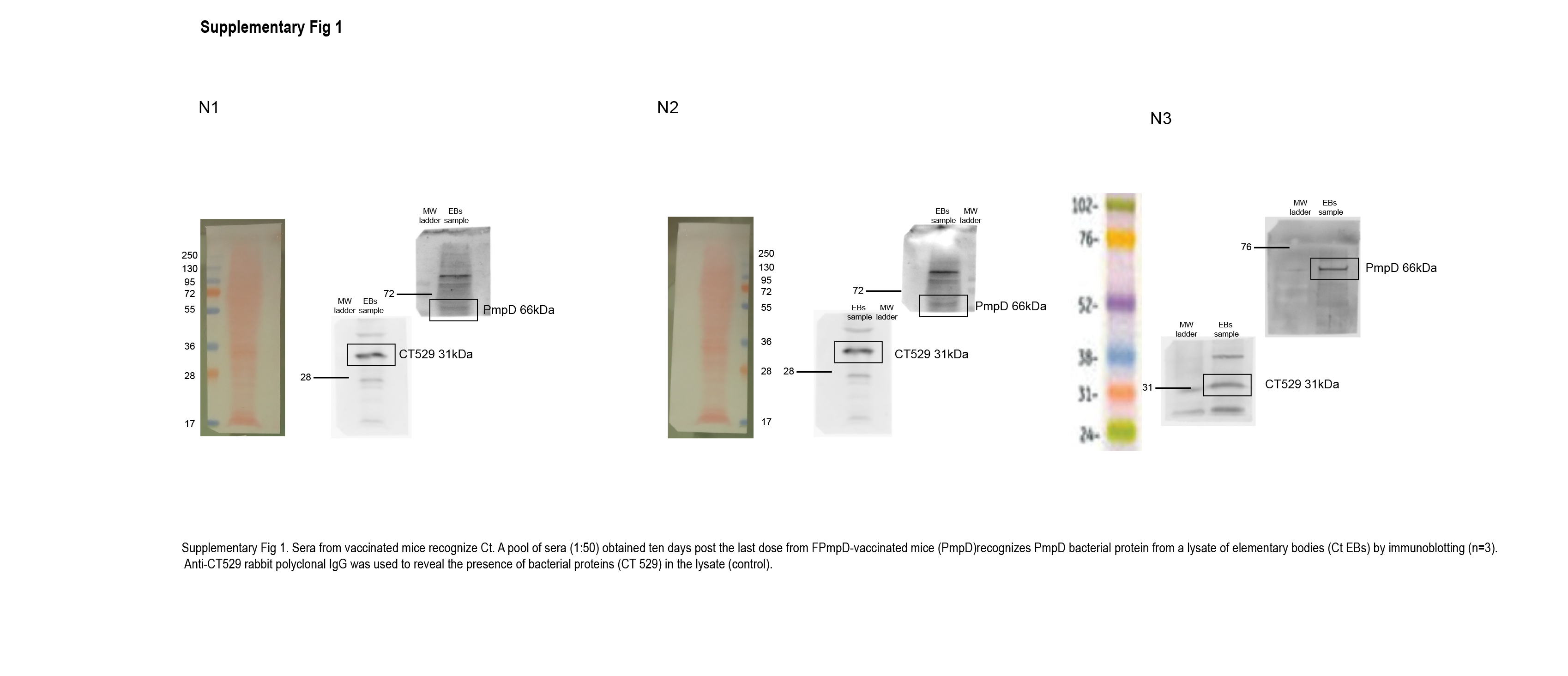

Supplement: Supplementary file 1 — Supplementary Information 1. [file 41598_2022_10633_MOESM1_ESM.tif]

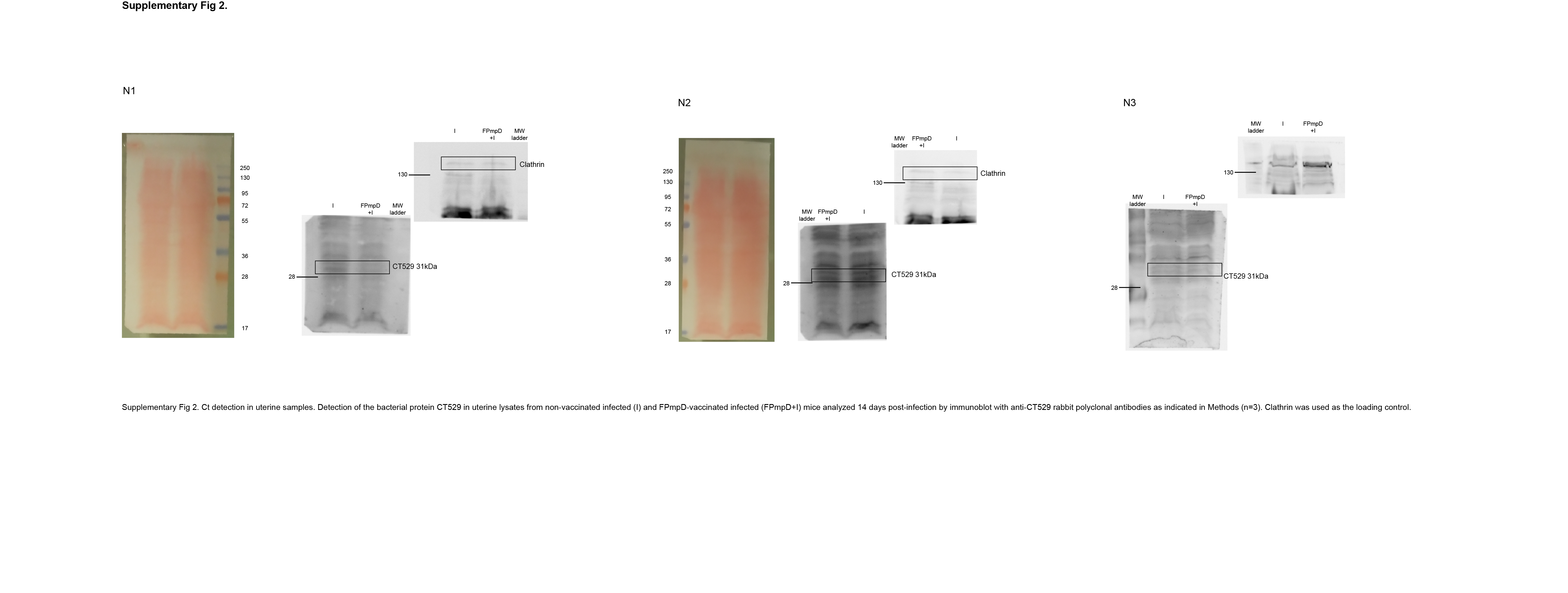

Supplement: Supplementary file 2 — Supplementary Information 2. [file 41598_2022_10633_MOESM2_ESM.tif]
